# Solubility and Thermodynamics Data of Cabozantinib Malate in Various Aqueous Solutions of Dimethyl Sulfoxide at Different Temperatures

**DOI:** 10.3390/molecules28237805

**Published:** 2023-11-27

**Authors:** Faiyaz Shakeel, Nazrul Haq, Sultan Alshehri, Ibrahim A. Alsarra

**Affiliations:** Department of Pharmaceutics, College of Pharmacy, King Saud University, P.O. Box 2457, Riyadh 11451, Saudi Arabia; nhaq@ksu.edu.sa (N.H.); salshehri1@ksu.edu.sa (S.A.); ialsarra@ksu.edu.sa (I.A.A.)

**Keywords:** antitumor drug, cabozantinib malate, DMSO, computational analysis, molecular interactions, cosolvent mixtures, solubility, thermodynamics

## Abstract

Cabozantinib malate (CBZM), a new anticancer medication, has been studied for its solubility and thermodynamic properties in a variety of {dimethyl sulfoxide (DMSO) + water (H_2_O)} mixtures at 298.2–318.2 K and 101.1 kPa. Using the shake flask technique, the solubility of CBZM was assessed and the results were correlated to the van’t Hoff, Apelblat, Buchowski–Ksiazczak *λh*, Yalkowsky–Roseman, Jouyban–Acree, and Jouyban–Acree-van’t Hoff models. There was a significant correlation between the experimental CBZM solubility data and all computational models, as evidenced by the error values for all computational models being less than 5.0%. Temperature and DMSO mass percentage improved the CBZM mole fraction solubility in the cosolvent solutions of {DMSO + H_2_O}. At 318.2 K, pure DMSO had the highest mole fraction solubility of CBZM (4.38 × 10^−2^), whereas pure H_2_O had the lowest mole fraction solubility (2.24 × 10^−7^ at 298.2 K). The positive values of computed thermodynamic parameters indicated that the dissolution of CBZM was endothermic and entropy-driven in all of the {DMSO + H_2_O} solutions investigated. It was found that the CBZM solvation in {DMSO + H_2_O} solutions is governed by enthalpy. When compared to CBZM-H_2_O, CBZM-DMSO showed the highest molecular interactions. The findings of this investigation demonstrated that DMSO has a great deal of potential for CBZM solubilization in H_2_O.

## 1. Introduction

**Figure 1:**
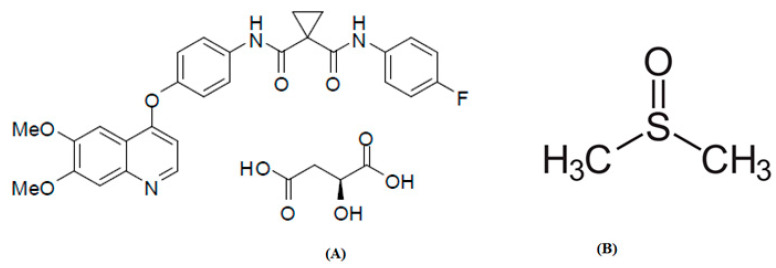
Chemical structures of (**A**) cabozantinib malate (CBZM) (taken from Ref. [2]) and (**B**) dimethyl sulfoxide (DMSO) (taken from https://en.wikipedia.org/wiki/Dimethyl_sulfoxide; accessed on 15 October 2023).

The prodrug cabozantinib malate (CBZM) breaks down into the active metabolite cabozantinib after metabolism [1,2]. Figure 1A shows the CBZM chemical structure [2]. It is a recently identified small molecule tyrosine kinase inhibitor (smTKI) that targets a particular tissue [1]. The smTKIs are utilized to treat cancer cells because they are overexpressed in malignant cells [3,4,5]. It has been suggested for the treatment of advanced renal carcinoma (ARC), castration-resistant prostate cancer (CRPC), medullary thyroid cancer (MTC), and hepatocellular carcinoma (HCC) [2]. It is offered for the treatment of MTC, CRPC, HCC, and ARC as commercial oral capsules (Cometriq^®^) or tablets (Cabometyx^®^) [2,6]. Commercial CBZM capsules and tablets have been found to have very low bioavailability in both animals and humans [2], which is likely a result of its poor solubility in an aqueous medium such as water (H_2_O). The design of CBZM formulations is difficult due to its poor water solubility. Poor bioavailability after oral administration and poor dissolution rate in dosage forms are the main difficulties of CBZM.

The importance of solubility data has long been recognized by the pharmaceutical industry [7,8]. By empowering chemists and scientists to make informed choices, the solubility data of pharmaceuticals, especially in the field of drug discovery and development, gives crucial knowledge to improve the quality of therapeutic compounds and increase the clinical success rate [9]. Furthermore, dose prediction is enhanced by using solubility data to predict in vivo pharmacokinetics [10,11]. The cosolvency technique is one of many that have been studied in pharmaceutical science and technology [11] to increase the solubility of therapeutic substances [12,13,14,15]. Dimethyl sulfoxide (DMSO) (Figure 1B) was used as a cosolvent in the cosolvency strategy to increase CBZM solubility in the present investigation. By using DMSO to boost CBZM solubility, a number of CBZM issues, including solubility, dissolution, absorption, and bioavailability issues, may be overcome. A crucial physicochemical property for many industrial processes, such as production, dosage form design, and other uses, is data on the solubility of a drug [16,17,18]. The solubility information for CBZM in mixtures of H_2_O and a cosolvent has not been adequately documented up to this point. CBZM has been found to be nearly insoluble in all aqueous solutions, including H_2_O [2]. It has been investigated to enhance the solubility, bioavailability, and therapeutic efficacy of CBZM using a number of lipophilic salts, lipid-based drug delivery strategies, and polymeric micelles [1,19]. We have previously described the solubility and thermodynamic data of CBZM at 298.2–318.2 K and 101.1 kPa in 12 different mono solvents of pharmaceutical significance, including H_2_O, methyl alcohol, ethyl alcohol, isopropyl alcohol, 1-butyl alcohol, 2-butyl alcohol, ethylene glycol, propylene glycol, polyethylene glycol–400, carbitol, ethyl acetate, and DMSO [20].

DMSO stock solution has been used as the *de facto* standard for several experiments, including the determination of a compound’s solubility and storage of various substances [21]. DMSO is also one of the most often applied solvents for solubility improvement as it is completely miscible with H_2_O in addition to low chemical reactivity [21,22]. DMSO showed low acute and chronic toxicity in animals [21,22,23]. It is not reported to be cytotoxic, carcinogenic, teratogenic and suitable as a cosolvent for drug solubilization [22,23]. However, at higher concentrations, it has been found to be cytotoxic [21,23]. The major disadvantage of DMSO use is that it has an impact on cell growth and enzyme activity [23]. Given that DMSO is known to interfere with protein–ligand interaction by altering solvent viscosity, it may have an impact on the drug’s in vivo pharmacokinetics [22,24]. According to reports, it lowers ligand–protein binding, which might improve the disposition of the drug’s kinetic profile [22]. Many poorly soluble drugs, such as raloxifene hydrochloride, sinapic acid, pyridazinone derivatives, baricitinib, meloxicam, clozapine, and isotretinoin, have become more soluble when DMSO is used as a potential solubilizer or cosolvent [18,25,26,27,28,29,30]. Regarding the solubility and thermodynamic data of CBZM in different {DMSO + H_2_O} mixes at varying temperatures and constant ambient/atmospheric pressure, there is no information available in the literature. This investigation was carried out to ascertain the solubility and thermodynamic characteristics of CBZM in various {DMSO + H_2_O} combinations, including neat DMSO and H_2_O, at 298.2–318.2 K under ambient pressure. The studied temperature range was selected randomly at the interval of 5.0 K. The temperature range of 298.2–318.2 K was maintained in such a manner that the maximum studied temperature (318.2 K) should not exceed the melting temperature of CBZM (i.e., 462.72 K) [20] and boiling temperatures of the studied solvents. DMSO and H_2_O have respective boiling temperatures of 462.2 K and 373.2 K. The highest temperature that was studied (318.2 K), was lower than the boiling points of DMSO and H_2_O as well as the melting temperature of CBZM. As a result, the present work’s temperature range remained within the previously stated range. Data collected during the study’s data collection phase may be useful for pre-formulation analysis, dosage form design, and the purification of the intended anticancer medicine.

## 2. Results and Discussion

### 2.1. CBZM Solid-Phase Characterization and Measured Solubility Data

To investigate the possibility of CBZM transforming into polymorphs or solvates/hydrates, CBZM solid-phase characterization prior to solubility assessment (pure CBZM) and post-solubility assessment (equilibrated CBZM) can be performed. In our earlier publication, we provide a full description of the results of this characterization on both samples of CBZM using differential scanning calorimetry (DSC) technology [20]. In our previously published work, it was discovered that the DSC thermograms of pure CBZM and equilibrated CBZM (recovered from methanol) were identical and displayed identical peak characteristics [20]. The CBZM sample that had been equilibrated also did not show any additional DSC signals. Since CBZM recovered from methanol showed no changes, it has been expected that CBZM from different aqueous mixtures of DMSO would not show any change in CBZM physical form. These results suggest that CBZM did not transform into solvates or hydrates or polymorphs. Table 1 lists the experimental CBZM solubility data at 298.2–318.2 K and 101.1 kPa in several aqueous DMSO solutions.

**Figure 2:**
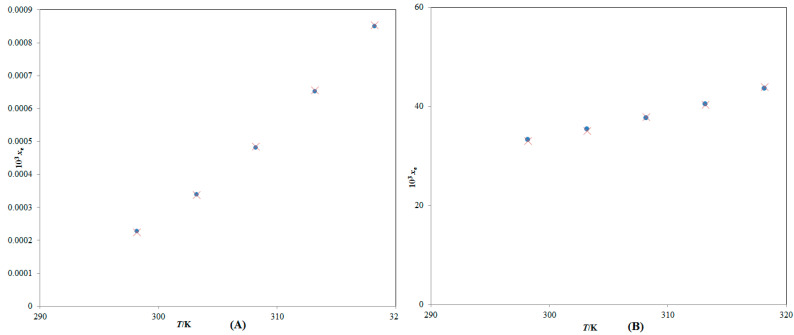
Graphic comparison between CBZM mole fraction solubility values (*x*_e_) in (**A**) neat H_2_O and (**B**) neat DMSO with those reported in the literature at 298.2–318.2 K. The symbol 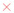 indicates the stated mole fraction solubilities of CBZM in (**A**) neat H_2_O and (**B**) neat DMSO, and the symbol 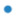 indicates the reported solubilities of CBZM in (**A**) neat H_2_O and (**B**) neat DMSO retrieved from Ref. [20].

**Figure 3:**
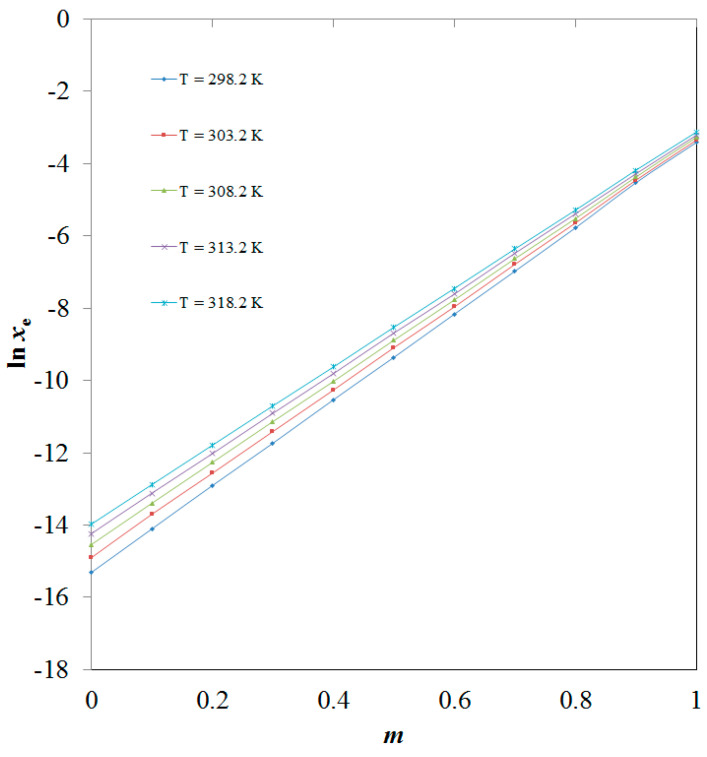
Effect of DMSO mass fraction (*m*) on logarithmic CBZM solubility values (ln *x*_e_) at 298.2–318.2 K.

There is no report on the solubility of CBZM in various aqueous mixtures of DMSO. However, CBZM mole fraction solubility values have been documented in pure DMSO and H_2_O at 298.2–318.2 K by our research group [20]. While, CBZM mole fraction solubility values have not been reported by other researchers. The mole fraction solubility is the ratio of the solute mole fractions to the sum of mole fractions of the solute and the solvents. Since it is expressed in mole fractions, it has no unit. Figure 2 shows a graphical comparison of the measured and reported solubility values of CBZM in pure DMSO and H_2_O at 298.2–318.2 K. The data presented in Figure 2 indicate a significant correlation between the experimentally determined and reported solubility values of CBZM in pure H_2_O and DMSO [20]. These results demonstrated that the experimentally determined solubility data from CBZM agreed well with the information that was previously published [20]. In general, it was found that the mole fraction solubilities of CBZM in pure DMSO and H_2_O were maximum and minimum, respectively. The reason why CBZM dissolves most completely in pure DMSO may be due to the weak polarity of DMSO as opposed to the strong polarity of H_2_O [28,29,30]. The higher solubility of CBZM in DMSO may also be due to intermolecular interactions between the -OH, C=O, and -MeO groups of CBZM (Figure 1A) and the S=O group of DMSO (Figure 1B). It has been found that CBZM has a pH-dependent solubility profile [2]. However, in the present study, pH-dependent solubility studies were not considered as the studies were performed in different mixtures of DMSO and H_2_O. Usually, pH-dependent solubility studies are performed using aqueous buffers of different pH to evaluate the pH-dependent solubility. Since, the buffers were not used in this study, these studies were not performed. The solubility of CBZM in various cosolvent combinations was enhanced by temperature and the mass fraction of DMSO. Between 298.2 and 318.2 K, the impact of DMSO mass percentage on CBZM solubility in logarithmic mole fractions was also investigated. The outcomes are documented in Figure 3. In all cosolvent solutions, the CBZM solubility rose linearly with the DMSO mass fraction at every temperature that was examined. Based on these results, CBZM can be considered as freely soluble in DMSO and practically insoluble in H_2_O. Due to this fact, DMSO was selected as the best solvent and H_2_O was selected as the antisolvent for CBZM. When compared to neat H_2_O, the solubility of CBZM in mole fractions increased significantly to neat DMSO. Hence, DMSO can be used as a cosolvent to solubilize CBZM in an aqueous medium such as H_2_O. Overall, DMSO can be utilized as a cosolvent in pre-formulation studies and dosage form design of CBZM especially in terms of liquid dosage forms. Due to the antisolvent property of H_2_O for CBZM, it can be employed in recrystallization studies of CBZM.

### 2.2. Assessment of Hansen Solubility Parameters (HSPs)

HSPs offer a quantitative assessment of the extent of solute–solvent interaction and may be a useful indicator of solubility or miscibility [31]. It is probable that solutes and solvents with comparable HSPs will dissolve into one another [32]. The identical HSPs also show that the solvent and solute have similar polarities. Thus, in this work, the HSPs of pure H_2_O, pure DMSO, and CBZM were estimated. Numerous applications of the HSPs estimation were found in diverse study fields [31,32]. The primary goal of the current effort was to obtain data regarding the solute and solvent’s solubility. Using Ref. [20], the total HSP (*δ*_t_) of the CBZM was obtained, and it was found to be 25.50 MPa^1/2^, suggesting weak polarity. Neat DMSO (*δ*_1_) and neat H2O (*δ*_2_) have HSP values of 23.60 MPa^1/2^ and 47.80 MPa^1/2^, respectively, according to the literature [20]. Other HSPs for CBZM, DMSO, and H_2_O have also been described and reported in our previously published work [20]. The range of HSP for numerous {DMSO + H_2_O} solutions devoid of CBZM (*δ*_mix_) was derived to be between 26.02 and 45.38 MPa^1/2^. It was discovered that when the mass percentage of DMSO increased, the *δ*_mix_ values in {DMSO + H_2_O} solutions decreased. As a result, the maximum and lowest *δ*_mix_ values were found at *m* = 0.1 and *m* = 0.9, respectively. However, it was found that lowering the *δ*_mix_ values improved the CBZM solubility values. The HSPs of CBZM (*δ*_t_ = 25.50 MPa^1/2^) and neat DMSO (*δ*_1_ = 23.60 MPa^1/2^) were often near to one other. The investigations also demonstrated that CBZM is more soluble in pure DMSO. The CBZM solubility data from experiments using combinations of {DMSO + H_2_O} therefore closely matched these findings.

### 2.3. Ideal Solubility (x_idl_) and Activity Coefficients (γ_i_) Data to Derive Molecular Interactions

The *x*_idl_ data for CBZM are listed in Table 1. At 298.2–318.2 K, the computed values for CBZM’s *x*_idl_ vary from 1.50 × 10^−3^ to 3.92 × 10^−3^. In comparison to experimental solubility (*x*_e_) values in pure H_2_O, CBZM showed significantly higher *x*_idl_ values. The *x*_e_ values of CBZM were greater than the *x*_idl_ values of pure DMSO at all temperatures investigated. The ideal cosolvent for CBZM solubilization can be employed since CBZM is more soluble in neat DMSO. The *γ*_i_ data for CBZM in numerous cosolvent solutions including pure solvents at 298.2–318.2 K are shown in Table 2. The CBZM’s *γ*_i_ value in neat H_2_O reached its greatest value at each of the investigated temperatures. However, the *γ*_i_ of CBZM was lowest in pure DMSO at each temperature taken into account. The *γ*_i_ values for CBZM were significantly lower for neat DMSO than for neat H_2_O. The lowest solubility of CBZM in H_2_O may account for the greatest *γ*_i_ for CBZM in pure H_2_O. According to these findings, the CBZM-DMSO combination exhibits a higher number of molecular solute–solvent interactions than the CBZM-H_2_O combination.

### 2.4. Correlation of CBZM Solubility Data

The solubility data of CBZM were correlated using six different computational approaches, including the van’t Hoff, Apelblat, Buchowski–Ksiazczak *λh*, Yalkowsky–Roseman, Jouyban–Acree, and Jouyban–Acree-van’t Hoff models [18,33,34,35,36,37,38,39,40,41]. The findings of the van’t Hoff model correlation are shown in Table 3. The overall root mean square deviation (*RMSD*) of this model was found to be 1.94%. The determination coefficient (*R*^2^) for CBZM was calculated to be in the range of 0.9928 to 0.9991 for all DMSO aqueous solutions as well as for pure solvents. There was a significant correlation between the van’t Hoff model predictions and the experimentally determined solubility data from the CBZM in all of the cosolvent solutions including pure solvents.

**Figure 4:**
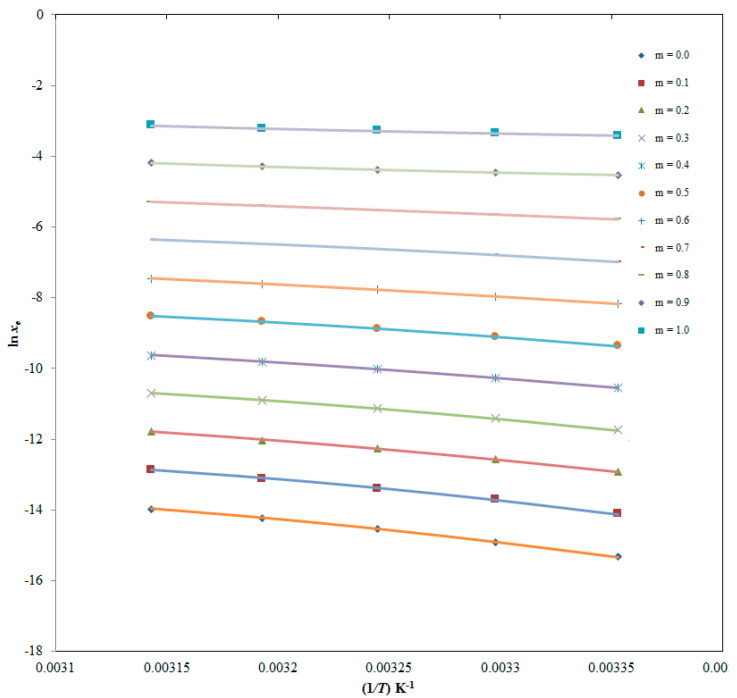
Graphical correlation of experimentally determined CBZM solubility values (*x*_e_) with the Apelblat model in various DMSO aqueous solutions (*m* = 0.0–1.0) as a function of 1/*T*; the symbols indicate CBZM *x*_e_ values and solid lines indicates the Apelblat model CBZM solubility values.

Figure 4 shows a graphical correlation between the experimental and Apelblat solubility values for CBZM in various cosolvent combinations including neat H_2_O, and DMSO. The results displayed in Figure 4 demonstrated a strong correlation between the Apelblat model and the experimentally determined solubility data of CBZM. Table 4 lists the Apelblat model parameters and correlation results for the CBZM in DMSO aqueous solutions including pure solvents. The overall *RMSD* of this model was determined to be 1.11%. CBZM revealed an *R*^2^ of 0.9989–0.9999 in all cosolvent combinations and pure DMSO and H_2_O. The Apelblat model results and the experimentally determined CBZM solubility data in various cosolvent combinations including neat solvents were also shown to be significantly correlated.

The results of the Buchowski–Ksiazaczak *λh* model correlation for CBZM in DMSO aqueous solutions and pure DMSO and H_2_O are shown in Table 5. The overall *RMSD* for this model was found to be 4.26%. The *R*^2^ for the CBZM was calculated to be between 0.9928 and 0.9991 for all DMSO aqueous solutions and neat solvents. Additionally, it was found that the Buchowski–Ksiazaczak *λh* model and the experimental solubility values from CBZM had a good degree of agreement.

In Table 6, the findings of the connection with the Yalkowsky–Roseman model are presented. This model’s overall *RMSD* was determined to be 2.98%, indicating that the Yalkowsky–Roseman model and *x*_e_ data for CBZM in numerous DMSO aqueous solutions are satisfactorily correlated.

The solubility value of CBZM was also associated to Jouyban–Acree and Jouyban–Acree-van’t Hoff models in a number of {DMSO + H_2_O} mixtures at various temperatures and solvent mixtures [41]. Table 7 presents the findings of the connection with the Jouyban–Acree and Jouyban–Acree-van’t Hoff models. The calculations show that the overall *RMSDs* for the Jouyban–Acree and Jouyban–Acree-van’t Hoff models are, respectively, 1.12% and 1.19%, indicating an exceptional correlation. Based on low *RMSD* values, all models generally demonstrated strong correlation. Nevertheless, it was not possible to compare the error values of each model with one another. All the error values of the models were within a narrow interval of the experimental uncertainties. This finding revealed that every model under investigation was able to replicate the experimental solubility data with the least possible error values. The semi-predictive Apelblat and van’t Hoff models correlate the solubility at various temperatures at the specified set of cosolvent combinations. At the specified set of temperatures, the solubility in the solvent mixtures is predicted by the Jouyban–Acree and Jouyban–Acree-van’t Hoff models. With the adjustable parameter fixed at zero, the Yalkowsky–Roseman model is a particular example of the Jouyban–Acree model. Because the Jouyban–Acree model uses the fewest adjustable parameters in comparison to other models, it performs the best among computational models that use adjustable parameters. When compared to other models with adjustable parameters, the Jouyban–Acree model is the most effective model for solubility correlation. The Yalkowsky–Roseman model, out of the six models examined, has a significant advantage because it does not call for any adjustable parameters. These findings led to the conclusion that the Yalkowsky–Roseman model, which has no adjustable parameters, is the best model for the correlation out of the six.

### 2.5. Thermodynamic Data for CBZM Dissolution

**Figure 5:**
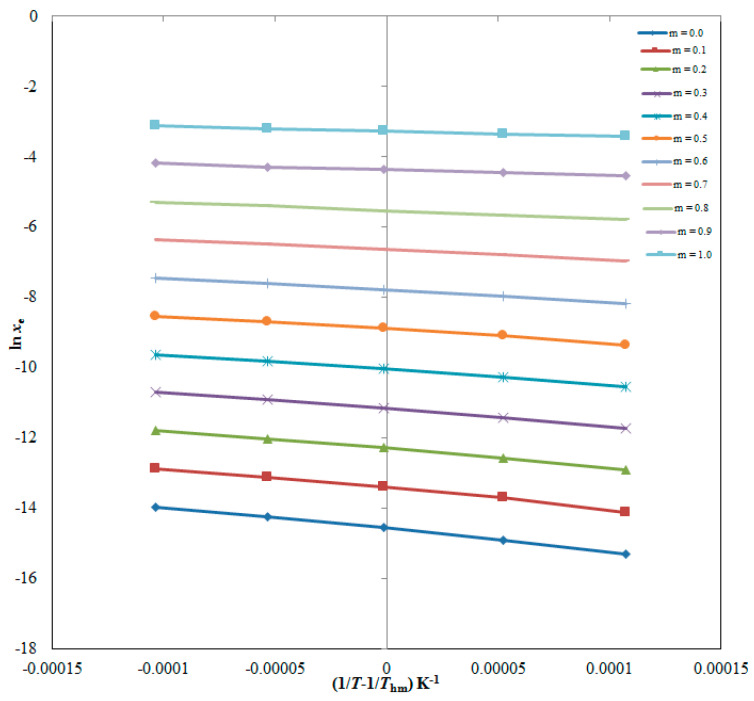
van’t Hoff graphs at the mean harmonic temperature (*T*_hm_) for CBZM graphed between ln *x*_e_ and 1/*T* − 1/*T*_hm_ for CBZM in numerous DMSO aqueous solutions (*m* = 0.0–1.0) to calculate various thermodynamic parameters.

The apparent standard enthalpy (Δ_sol_*H*°) data for CBZM in each DMSO aqueous solution as well as pure solvents were calculated using the van’t Hoff approach. Figure 5 shows the linear van’t Hoff curves of CBZM in all DMSO aqueous solutions, as well as in pure DMSO and H_2_O, where *R*^2^ > 0.99 was anticipated, as stated in Table 8. The outcomes for all thermodynamic parameters are also shown in Table 8. CBZM Δ_sol_*H*° data in numerous cosolvent mixtures and neat solvents ranged from 11.43 to 52.71 kJ mol^−1^. CBZM apparent standard Gibbs energy (Δ_sol_*G*°) data in numerous DMSO aqueous solutions and neat solvents were ranged from 8.38 to 37.36 kJ mol^−1^. These data for CBZM’s Δ_sol_*H*° and Δ_sol_*G*° indicated endothermic dissolution of CBZM in numerous DMSO aqueous solutions including pure solvents [18,25]. CBZM apparent standard entropy (Δ_sol_*S*°) data between 9.05 and 49.82 J mol^−1^ K^−1^ were recorded in distinct cosolvent mixtures and neat solvents, indicating that entropy-driven CBZM dissolution held in these cosolvent combinations and neat solvents [18]. Finally, it has been revealed that CBZM dissolution was endothermic and entropy-driven in all cosolvent combinations, including neat solvents [18,25].

### 2.6. Enthalpy–Entropy Compensation Analyses

**Figure 6:**
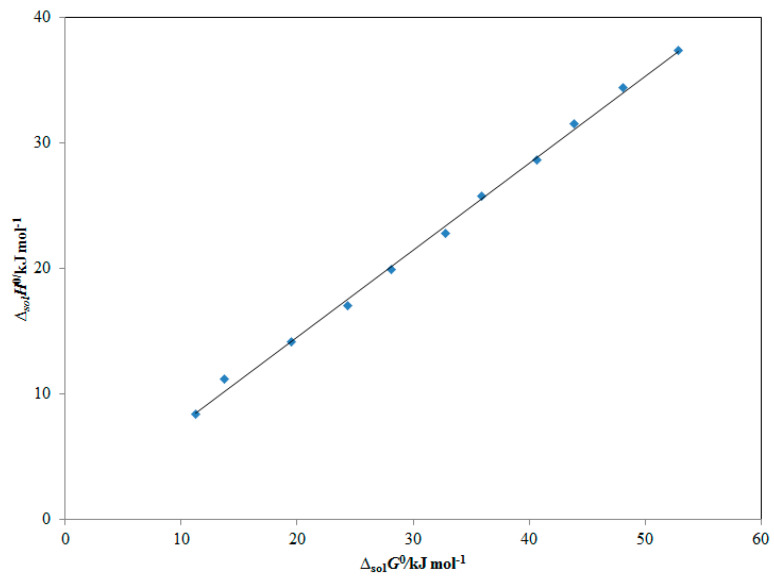
Apparent standard enthalpy **(**Δ_sol_*H°*) vs. apparent standard Gibbs energy (Δ_sol_*G°*) enthalpy–entropy compensation analyses for CBZM solubility in numerous DMSO aqueous solutions (*m* = 0.0–1.0) at *T*_hm_ = 308 K.

To examine the solvation behavior of CBZM in various cosolvent combinations as well as neat solvents, an enthalpy–entropy compensation analyses was used. The findings are shown in Figure 6. In all cosolvent combinations and pure solvents, Figure 6 shows that CBZM produces a linear Δ_sol_*H*° vs. Δ_sol_*G*° graph with a slope >1.0 and an *R*^2^ > 0.99. The CBZM solvation-driven process is projected to be enthalpy-driven in all cosolvent combinations and pure solvents based on these findings. The fact that CBZM solvates more effectively in pure DMSO molecules than in pure H_2_O molecules should be used to explain this CBZM solvation process [18,25]. As a result, CBZM-DMSO molecules interacted with one another more strongly than CBZM-H_2_O molecules. In several DMSO aqueous solutions as well as in neat solvents, CBZM solvated in the same manner as reported for raloxifene hydrochloride, sinapic acid, pyridazinone derivative, baricitinib, and isotretinoin [18,25,26,27,30].

## 3. Materials and Methods

### 3.1. Materials

CBZM was provided by Beijing Mesochem Technology (Beijing, China). DMSO was provided by E-Merck (Darmstadt, Germany). Purified/deionized water was obtained from Milli-Q unit. The aggregated information of each material is included in Table 9.

### 3.2. Measurement of CBZM Solubility in DMSO Aqueous Solutions and Pure Solvents

A digital analytical balance (Mettler Toledo, Greifensee, Switzerland) with a sensitivity of 0.10 mg was used to measure the mass of each cosolvent mixture. A series of DMSO aqueous solutions (*m* = 0.10–0.90), were investigated. Three replications of each cosolvent mixture were taken [18]. CBZM’s solubility values in numerous DMSO aqueous solutions (*m* = 0.0–1.0) and pure solvents were determined utilizing a shake flask methodology at five distinct temperature and fixed atmospheric pressure [42]. Essentially, extra CBZM crystals were mixed with known amounts of each cosolvent mixture and neat solvents in triplicates. All the mixtures were vortexed for about 5 min. To achieve equilibrium, the resulting mixes were transferred to a WiseBath WSB Shaking Water Bath (Model WSB-18/30/-45, Daihan Scientific Co. Ltd., Seoul, Republic of Korea) for 72 h at 100 rpm for continuous shaking [20]. After the samples reached equilibrium, they were removed from the shaker and centrifuged at 5000 rpm for 30 min at 298.2 K. The uncertainty in water bath temperature was recorded as 0.15 K. The equilibrium time of 72 h was optimized by preliminary experiments. Under preliminary experiments, CBZM solubility was measured at 24, 48, 48, 72, and 96 h. It was observed that CBZM solubility was not changed considerably after 72 h, and hence 72 h was selected as the equilibrium time. After the supernatants were separated and diluted with mobile phase (if needed), the CBZM content was evaluated at 244 nm using a previously described HPLC technique [20]. By using their standard formulae found in the literature [25,26,27], CBZM *x*_e_ values were obtained.

### 3.3. HSPs of CBZM and Various DMSO Aqueous Solutions

The degree to which a drug dissolves in a pure solvent or aqueous cosolvent solutions directly correlates to its HSP. When a drug’s HSP is close to that of a certain solvent, it supposedly has the greatest solubility in that solvent [31]. The HSPs for the chosen drug CBZM, pure DMSO, and H_2_O were consequently computed. The values for CBZM, pure H_2_O, and pure DMSO were obtained from reference [20].

With the help of Equation (1), the *δ*_mix_ was derived [43]:

(1) $$\delta_{\mathrm{mix}}=\alpha \delta_{1}+\left(1-\alpha \right)\delta_{2}$$

where *α* is the volume fraction of DMSO in DMSO aqueous solutions.

### 3.4. CBZM x_idl_ and γ_i_ Data to Derive Molecular Interactions

With the help of Equation (2), the *x*_idl_ of CBZM at 298.2–318.2 K was derived [44]:

(2) $$\ln\;x_{\mathrm{idl}}=\frac{-\Delta H_{\mathrm{fus}}\left(T_{\mathrm{fus}}-T\right)}{RT_{\mathrm{fus}}T}+\left(\frac{\Delta C_{p}}{R}\right)[\frac{T_{\mathrm{fus}}-T}{T}+\ln\left(\frac{T}{T_{\mathrm{fus}}}\right)]\;$$

where *T* = absolute temperature, *T*_fus_ = CBZM fusion/melting temperature, *R* = universal gas constant, Δ*H*_fus_ = CBZM fusion enthalpy, and Δ*C*_p_ = the difference between the molar heat capacities of CBZM in its solid and liquid states [45].

The data for *T*_fus_, Δ*H*_fus_, and Δ*C*_p_ for CBZM were collected from Ref. [20], and they are 462.72 K, 56.93 kJ mol^−1^, and 123.03 J mol^−1^ K^−1^, respectively. Now, Equation (2) was used to determine the *x*_idl_ values for CBZM. The *γ*_i_ values for CBZM in all cosolvent combinations and pure solvents were obtained using Equation (3) [44,46]:

(3) $$\gamma_{i}=\frac{x_{\mathrm{idl}}}{x_{e}}$$

The chemical foundations of molecular interactions between the solute and solvent were characterized using CBZM *γ*_i_ data.

### 3.5. Computational Analysis

Computational validation of experimentally determined solubility data is necessary for meaningful forecasts and validations [33,34]. Six distinct computational models, namely van’t Hoff, Apelblat, Buchowski–Ksiazczak *λh*, Yalkowsky–Roseman, Jouyban–Acree, and Jouyban–Acree-van’t Hoff models were utilized to correlate the experimental solubility data from CBZM [18,33,34,35,36,37,38,39,40,41]. By Equation (4), van’t Hoff model solubility (*x*^van’t^) of CBZM in cosolvent combinations and pure solvents was derived [18]:

(4) $$\ln\;x^{\mathrm{van}’t}=a+\frac{b}{T}$$

where *a* and *b* are Equation (4) model coefficients derived by the least squares approach [39]. The data of *x*_e_ and *x*^van’t^ for the CBZM were correlated using the *RMSD.* A formula that was derived from the literature was used to determine the *RMSD* [47]. By Equation (5), the Apelblat model solubility (*x*^Apl^) of CBZM in cosolvent combinations and pure solvents was derived [35,36]:

(5) $$\ln\;x^{\mathrm{Apl}}=A+\frac{B}{T}+C\ln\left(T\right)$$

where the Equation (5) model coefficients were obtained using nonlinear multiple regression analysis based on the experimental CBZM solubility values displayed in Table 1 [47]. The outcomes from CBZM’s *x*_e_ and *x*^Apl^ were connected in terms of *RMSD*. Utilizing Equation (6), the Buchowski–Ksiazczak *λh* solubility (*x*^λh^) of CBZM in cosolvent mixtures and neat solvents was derived [37,38]:

(6) $$\ln\;[1+\frac{\lambda \left(1-x^{\lambda h}\right)}{x^{\lambda h}}]\;=\;\lambda h\;[\frac{1}{T}-\frac{1}{T_{\mathrm{fus}}}]$$

where, *λ* and *h* are Equation (6) model coefficients.

Equations (4)–(6) cannot be utilized to predict the solubility data of a binary solvent combination at different solvent compositions since they reflect solubility data at different temperatures in a particular solvent composition [41,48,49]. Cosolvency models such as the Yalkowsky–Roseman, Jouyban–Acree, and Jouyban–Acree-van’t Hoff models are needed to produce these forecasts. By Equation (7), the logarithmic solubility of Yalkowsky–Roseman model (log *x*^Yal^) for CBZM in numerous cosolvent compositions was derived [40]:

(7) $$\log x^{\mathrm{Yal}}=w_{1}\log x_{1}+w_{2}\log x_{2}$$

where *x*_1_ = CBZM solubility in DMSO; *x*_2_ = CBZM solubility in H_2_O; *w*_1_ = DMSO mass fraction, and *w*_2_ = H_2_O mass fraction. Equation (7) links data on drug solubility in various solvent mixtures at a specific temperature.

The Jouyban–Acree model used Equation (8) to determine the solubility of medicines in various cosolvent mixes and temperature ($x_{m,T}$) [41]:

(8) $$\ln x_{m,T}=w_{1}\ln x_{1,T}+w_{2}\ln x_{2,T}+(\frac{w_{1}.w_{2}}{T})\sum_{i=0}^{2}J_{i}{\left(w_{1}-w_{2}\right)}^{i}$$

where $x_{1,T}$ and $x_{2,T}$ are CBZM solubility in DMSO and H_2_O and *J_i_* is the Equation (8) model coefficient. By putting the *J_i_* value, the trained version of Equation (8) for the present dataset can be expressed using Equation (9):

(9) $$\ln\;x_{m}{,}_{T}\;=w_{1}\ln x_{1}+w_{2\;}\ln\;x_{2}+\frac{40,476w_{1}\;w_{2}}{T}$$

The CBZM solubility values in neat DMSO and H_2_O must be utilized as input data when calculating the CBZM solubility in cosolvent solutions at the target temperature. Equations (4) and (8) can be used to produce the Jouyban–Acree-van’t Hoff model (Equation (10)) to get around this limitation [41]:

(10) $$\ln\;x_{m}{,}_{T}=w_{1}\left(A_{1}+\frac{B_{1}}{T}\right)+w_{2}\;\left(A_{2}+\frac{B_{2}}{T}\right)+\left[\frac{w_{1}w_{2}}{T}\;\overset{2}{\underset{i=0}{\sum }}J_{i}\left(w_{1}-w_{2}\right)\right]$$

where *A*_1_, *B*_1_, *A*_2_, *B*_2_, and *J_i_* are the model parameters of Equation (10). For the present data set, the trained version of Equation (10) can be expressed using Equation (11):

(11) $$\ln\;x_{m}{,}_{T}=w_{1}\left(1.0960-\frac{1346.7}{T}\right)+w_{2}\;\left(6.0050-\frac{6345.1}{T}\right)+\frac{38,476w_{1}w_{2}}{T}$$

The adjustable parameters of all models were determined using MS Excel 2016 program.

### 3.6. Apparent Thermodynamic Analyses

All of the CBZM’s apparent thermodynamic properties were determined at *T*_hm_ [44]. The reported equation was used to determine the *T*_hm_ [41,44]. Then, 308 K was calculated as the *T*_hm_ for CBZM. Several thermodynamic parameters were obtained via an apparent thermodynamic analysis. These parameters were computed using the van’t Hoff and Gibbs equations. Using Equation (12) and *T*_hm_ = 308 K, the Δ_sol_*H*^0^ data for CBZM in cosolvent mixtures and pure solvents were calculated [44,50]:

(12) ∂ln xe∂1T−1ThmP=−ΔsolH0R

The Δ_sol_*H*^0^ for CBZM was obtained by the plotted van’t Hoff curves between ln *x*_e_ of CBZM and 1T−1Thm. The van’t Hoff curves for CBZM in cosolvent compositions and pure solvents are displayed in Figure 5.

Furthermore, at *T*_hm_ = 308 K, the Δ_sol_*G*^0^ for CBZM in cosolvent combinations and pure solvents was derived utilizing the Krug et al. approach by Equation (13) [50]:

(13) $$\Delta_{\mathrm{sol}}G^{0}=-RT_{\mathrm{hm}}\times \mathrm{intercept}\;\;\;\;\;\;\;\;\;\;\;\;\;\;\;\;\;\;$$

where the intercept values for CBZM in cosolvent combinations and pure solvents were calculated using the van’t Hoff plots depicted in Figure 5.

By Equation (14), the Δ_sol_*S*^0^ for CBZM in cosolvent mixtures and neat solvents was derived [44,50,51]:

(14) $$\Delta_{\mathrm{sol}}S^{0}=\frac{\Delta_{\mathrm{sol}}H^{0}-\Delta_{\mathrm{sol}}G^{0}}{T_{\mathrm{hm}}}\;\;\;\;\;\;\;\;\;\;\;\;\;\;\;\;\;\;\;\;\;\;\;\;\;\;\;\;\;\;\;\;\;$$

### 3.7. Enthalpy-Entropy Compensation Analyses

An enthalpy–entropy compensation analyses was performed, as previously described [18], to assess the solvation behavior of CBZM in cosolvent mixtures and neat solvents. Weighted curves of Δ_sol_*H*° vs. Δ_sol_*G*° were constructed at *T*_hm_ = 308 K for this experiment [52,53].

## 4. Conclusions

The solubility data of CBZM has not yet been reported in any of the {DMSO + H_2_O} combinations. This investigation examined the solubility of CBZM at varied temperatures and fixed pressures in numerous DMSO aqueous solutions including pure solvents. The CBZM solubility values were fluctuated with temperature and DMSO mass percentage in all cosolvent combinations, including pure solvents. The solubilities of CBZM in pure DMSO and H_2_O were found to be the highest and lowest for each investigated temperature, respectively. For all cosolvent combinations, including pure solvents, there was a good agreement between experimentally determined CBZM solubility data and six different computer models. In various cosolvent combinations as well as in pure solvents, it was found that all thermodynamic data, including Δ_sol_*H°*^,^ Δ_sol_*G°*, and Δ_sol_*S°*, were positive, indicating endothermic and entropy-driven CBZM dissolution. Both in pure solvents and in all cosolvent combinations, enthalpy drove the CBZM solvation process. For pre-formulation evaluation, recrystallization, purification, and dosage form design for the CBZM, the information gained from this study may be helpful.

## Figures and Tables

**Table 1:** Experimental (*x*_e_) and ideal solubility (*x***_idl_**) values of CBZM in several DMSO aqueous solutions (DMSO mass fraction *m* = 0.0–1.0) at 298.2–318.2 K and 101.1 kPa.

*m* ^a^	*x*_e_^b^				
	*T* = 298.2 K	*T* = 303.2 K	*T* = 308.2 K	*T* = 313.2 K	*T* = 318.2 K
0.0	2.24 × 10^−7^	3.37 × 10^−7^	4.85 × 10^−7^	6.55 × 10^−7^	8.53 × 10^−7^
0.1	7.46 × 10^−7^	1.12 × 10^−6^	1.52 × 10^−6^	2.01 × 10^−6^	2.55 × 10^−6^
0.2	2.48 × 10^−6^	3.48 × 10^−6^	4.69 × 10^−6^	6.00 × 10^−6^	7.57 × 10^−6^
0.3	8.00 × 10^−6^	1.11 × 10^−5^	1.46 × 10^−5^	1.83 × 10^−5^	2.25 × 10^−5^
0.4	2.66 × 10^−5^	3.48 × 10^−5^	4.42 × 10^−5^	5.49 × 10^−5^	6.59 × 10^−5^
0.5	8.61 × 10^−5^	1.12 × 10^−4^	1.38 × 10^−4^	1.69 × 10^−4^	1.98 × 10^−4^
0.6	2.48 × 10^−4^	3.47 × 10^−4^	4.21 × 10^−4^	4.95 × 10^−4^	5.79 × 10^−4^
0.7	9.33 × 10^−4^	1.12 × 10^−3^	1.31 × 10^−3^	1.53 × 10^−3^	1.72 × 10^−3^
0.8	3.09 × 10^−3^	3.53 × 10^−3^	3.99 × 10^−3^	4.55 × 10^−3^	5.04 × 10^−3^
0.9	1.07 × 10^−2^	1.15 × 10^−2^	1.26 × 10^−2^	1.38 × 10^−2^	1.52 × 10^−2^
1.0	3.29 × 10^−2^	3.50 × 10^−2^	3.78 × 10^−2^	4.02 × 10^−2^	4.38 × 10^−2^
*x*_**idl**_	1.50 × 10^−3^	1.91 × 10^−3^	2.44 × 10^−3^	3.10 × 10^−3^	3.92 × 10^−3^

^a^ The uncertainties *u* are *u*(*T*) = 0.15 K, *u*(*m*) = 0.0007, and *u*(*p*) = 2 kPa, and ^b^ the relative uncertainty *u*_r_ in solubility is *u*_r_(*x*_e_) = 0.05.

**Table 2:** Activity coefficients (*γ_i_*) of CBZM in distinct DMSO aqueous solutions (*m* = 0.0–1.0) at 298.2–318.2 K.

*m*	*γ*_i_				
	*T* = 298.2 K	*T* = 303.2 K	*T* = 308.2 K	*T* = 313.2 K	*T* = 318.2 K
0.0	6703	5689	5044	4739	4597
0.1	2011	1712	1608	1542	1538
0.2	605.5	550.8	521.3	517.5	518.0
0.3	187.7	173.5	167.9	169.7	174.4
0.4	56.40	55.14	55.29	56.55	59.49
0.5	17.42	17.07	17.69	18.40	19.80
0.6	5.281	5.524	5.800	6.266	6.776
0.7	1.609	1.699	1.853	2.023	2.267
0.8	0.4851	0.5430	0.6120	0.6808	0.7771
0.9	0.1392	0.1658	0.1932	0.2245	0.2572
1.0	0.0455	0.0547	0.0646	0.0771	0.0894

**Table 3:** Findings for the van’t Hoff model including model coefficients (*a* and *b*), *R*^2^, and *RMSD* for CBZM in various DMSO aqueous solutions (*m* = 0.0–1.0).

*m*	*a*	*b*	*R* ^2^	Overall *RMSD* (%)
0.0	6.0050	–6345.1	0.9951	
0.1	5.3286	–5781.7	0.9928	
0.2	4.8131	–5275.2	0.9961	
0.3	4.6768	–4884.5	0.9946	
0.4	3.9428	–4311.2	0.9972	
0.5	3.8641	–3935.2	0.9949	1.94
0.6	3.1637	–3375.2	0.9984	
0.7	2.8568	–2927.5	0.9961	
0.8	2.0735	–2340.7	0.9991	
0.9	0.99780	–1651.9	0.9946	
1.0	1.0960	–1346.7	0.9952	

**Table 4:** Findings of the Apelblat model including model coefficients (*A*, *B*, and *C*), *R*^2^, and *RMSD* for CBZM in various DMSO aqueous solutions (*m* = 0.0–1.0).

*m*	*A*	*B*	*C*	*R* ^2^	Overall *RMSD* (%)
0.0	1171.2	–59,844	–173.03	0.9996	
0.1	1292.7	–64,886	–191.17	0.9995	
0.2	854.93	–44,308	–126.23	0.9995	
0.3	945.45	–48,077	–139.70	0.9995	
0.4	617.15	–32,468	–91.057	0.9999	
0.5	758.16	–38,566	–112.00	0.9998	1.11
0.6	337.58	–18,734	–49.657	0.9996	
0.7	497.02	–25,617	–73.381	0.9999	
0.8	132.99	–8356.9	–19.439	0.9995	
0.9	–314.18	12,808	46,808	0.9998	
1.0	–217.67	8689.8	32.489	0.9989	

**Table 5:** Findings of Buchowski–Ksiazaczak *λh* model for CBZM in various DMSO aqueous solutions (*m* = 0.0–1.0).

*m*	*λ*	*h*	*R* ^2^	Overall *RMSD* (%)
0.0	6.7074	946.01	0.9951	
0.1	6.1660	937.72	0.9928	
0.2	5.8575	900.57	0.9961	
0.3	4.8790	1001.1	0.9945	
0.4	4.3741	985.64	0.9972	
0.5	3.6405	1080.9	0.9949	4.26
0.6	3.1306	1078.1	0.9984	
0.7	2.4700	1185.2	0.9961	
0.8	1.9852	1179.0	0.9991	
0.9	1.5723	1050.6	0.9946	
1.0	0.81440	1653.4	0.9952	

**Table 6:** Findings of Yalkowsky–Roseman model for CBZM in numerous DMSO aqueous solutions (*m* = 0.1–0.9) at 298.2–318.2 K.

*m*	Log *x*^Yal^					Overall *RMSD* (%)
	*T* = 298.2 K	*T* = 303.2 K	*T* = 308.2 K	*T* = 313.2 K	*T* = 318.2 K	
0.1	−6.13	−5.97	−5.82	−5.70	−5.59	
0.2	−5.61	−5.46	−5.33	−5.22	−5.12	
0.3	−5.09	−4.96	−4.84	−4.74	−4.65	
0.4	−4.58	−4.46	−4.35	−4.26	−4.18	
0.5	−4.06	−3.96	−3.86	−3.78	−3.71	2.98
0.6	−3.54	−3.46	−3.37	−3.30	−3.24	
0.7	−3.03	−2.96	−2.89	−2.82	−2.77	
0.8	−2.51	−2.45	−2.40	−2.34	−2.30	
0.9	−1.99	−1.95	−1.91	−1.86	−1.82	

**Table 7:** Findings of Jouyban–Acree and Jouyban–Acree-van’t Hoff models for CBZM in different {DMSO + H_2_O} mixtures.

System	Jouyban–Acree	Jouyban–Acree-Van’t Hoff
		*A*_1_ 1.0960 *B*_1_ –1346.7 *A*_2_ 6.0050 *B*_2_ –6345.1 *J*_i_ 38,476 1.19
{DMSO + H_2_O}	*J*_i_ 40,476	
	
	
*RMSD* (%)	1.12	

**Table 8:** Apparent thermodynamic parameters (Δ_sol_*H*^0^, Δ_sol_*G*^0^, and Δ_sol_*S*^0^) along with *R*^2^ values for CBZM in numerous DMSO aqueous solutions (*m* = 0.0–1.0) ^c^.

*m*	Δ_sol_*H*^0^/kJ mol^−1^	Δ_sol_*G*^0^/kJ mol^−1^	Δ_sol_*S*^0^/J mol^−1^ K^−1^	*R* ^2^
0.0	52.71	37.36	49.82	0.9952
0.1	48.03	34.41	44.21	0.9929
0.2	43.82	31.52	39.92	0.9961
0.3	40.57	28.62	38.80	0.9946
0.4	35.81	25.74	32.71	0.9973
0.5	32.78	22.81	32.35	0.9946
0.6	28.02	19.95	26.18	0.9985
0.7	24.18	17.03	23.21	0.9963
0.8	19.48	14.15	17.30	0.9991
0.9	31.98	11.19	9.05	0.9957
1.0	11.43	8.38	9.91	0.9973

^c^ The relative uncertainties are *u*(Δ_sol_*H*^0^) = 0.043, *u*(Δ_sol_*G*^0^) = 0.042, and *u*(Δ_sol_*S*^0^) = 0.047.

**Table 9:** Details of materials utilized in the experiments.

Material	Molecular Formula	Molar Mass (g mol^−1^)	CAS RN	Purification Method	Mass Fraction Purity	Analysis Method	Source
CBZM	C_32_H_30_FN_3_O_10_	635.60	4759-48-2	None	>0.99	HPLC	Beijing Mesochem
DMSO	C_2_H_6_OS	78.13	67-68-5	None	>0.99	GC	E-Merck
Water	H_2_O	18.07	7732-18-5	None	-	-	Milli-Q

## Data Availability

The data are available on reasonable request from corresponding author.

## Nomenclature

CBZM	Cabozantinib malate
DMSO	Dimethyl sulfoxide
H_2_O	Water
smTKI	Small molecule tyrosine kinase inhibitor
ARC	Advanced renal carcinoma
CRPC	Castration-resistant prostate carcinoma
MTC	Medullary thyroid cancer
HCC	Hepatocellular carcinoma
DSC	Differential scanning calorimetry
*m*	DMSO mass fraction in {DMSO + H_2_O} mixtures
*T*	Absolute temperature (K)
*u* *(T)*	Uncertainty in temperature (K)
*u* *(m)*	Uncertainty in DMSO mass fraction
*u* *(p)*	Uncertainty in atmospheric pressure (kPa)
*x*_e_	Experimental mole fraction solubility of CBZM
*U(x*_e_ *)*	Relative uncertainty in CBZM solubility
*x*_idl_	Ideal solubility of CBZM in mole fraction
HSP	Hansen solubility parameter
*δ*_t_	Total HSP for CBZM (MPa^1/2^)
*δ*_1_	HSP of neat DMSO (MPa^1/2^)
*δ*_2_	HSP of neat water (MPa^1/2^)
*δ*_mix_	HSP for {DMSO + H_2_O} mixtures free of CBZM (MPa^1/2^)
*α*	DMSO volume fraction in {DMSO + H_2_O} mixtures
*γ*_i_	Activity coefficient of CBZM
*R* ^2^	Correlation coefficient for CBZM
*RMSD*	Root mean square deviations (%)
*a* and *b*	Parameters of the van’t Hoff model
*A, B,* and *C*	Parameters of the Apelblat model
*λ* and *h*	Parameters of the Buchowski–Ksiazaczak *λh* model
*J*_i_	Parameter of the Jouyban–Acree model
*A*_1_*, B*_1_*, A*_2_ and *B*_2_	Parameter of the Jouyban–Acree-Van’t Hoff model
*x* ^Van’t^	Van’t Hoff model solubility of CBZM
*x* ^Apl^	Apelblat model solubility of CBZM
*x* ^Yal^	Yalkowsky model solubility of CBZM
*x*_m,T_	Jouyban–Acree model solubility of CBZM
Δ_sol_*H*^0^	Apparent standard enthalpy of CBZM (kJ mol^−1^)
Δ_sol_*G*^0^	Apparent standard Gibbs energy of CBZM (kJ mol^−1^)
Δ_sol_*S*^0^	Apparent standard entropy of CBZM (J mol^−1^ K^−1^)
*u*(Δ_sol_*H*^0^)	Relative uncertainty in Δ_sol_*H*^0^
*u*(Δ_sol_*G*^0^)	Relative uncertainty in Δ_sol_*G*^0^
*u*(Δ_sol_*S*^0^)	Relative uncertainty in Δ_sol_*S*^0^
*T*_hm_	Mean harmonic temperature (K)
*T*_fus_	CBZM fusion temperature (K)
*R*	Universal gas constant (J mol^−1^ K^−1^)
Δ*H*_fus_	CBZM molar fusion enthalpy (kJ mol^−1^)
Δ*C*_p_	Difference in molar heat capacity of CBZM (J mol^−1^ K^−1^)
*x*_1_	Mole fraction of CBZM in neat DMSO
*x*_2_	Mole fraction of CBZM in neat H_2_O
*w*_1_	DMSO mass fraction
*w*_2_	H_2_O mass fraction
